# Injectable Matrix Metalloproteinase-Responsive Polypeptide Hydrogels as Drug Depots for Antitumor Chemo-Immunotherapy

**DOI:** 10.3390/pharmaceutics17111453

**Published:** 2025-11-11

**Authors:** Shuang Liang, Tianran Wang, Junfeng Ding, Jiaxuan Yang, Chaoliang He, Yan Rong

**Affiliations:** 1 State Key Laboratory of Polymer Science and Technology, Changchun Institute of Applied Chemistry, Chinese Academy of Sciences, Changchun 130022, China; 2 School of Applied Chemistry and Engineering, University of Science and Technology of China, Hefei 230026, China

**Keywords:** injectable hydrogel, MMP-responsive, chemo-immunotherapy, drug sustained release

## Abstract

**Background:** The potential of injectable hydrogels as drug depots lies in their ability to achieve local and sustained co-delivery of chemotherapeutic drugs and immunostimulants for combined tumor therapy. **Method:** In this study, we devised a localized chemo-immunotherapeutic strategy by co-loading the chemotherapeutic drug, oxaliplatin (OXA), and the immune-checkpoint blockade (ICB) antibody, anti-programmed cell death protein ligand 1 (anti-PD-L1), into a matrix metalloproteinase (MMP)-responsive injectable poly(L-glutamic acid) hydrogel (MMP-gel). **Results:** The in situ gelation of hydrogels enables local retention of OXA and model antibody IgG, as well as MMP-triggered sustained release. Meanwhile, the OXA-loaded MMP-gel caused the immunogenic cell death (ICD) of tumor cells. When administered intratumorally in mice carrying B16F10 melanoma, the MMP-gel co-loaded with OXA and anti-PD-L1 (OXA&anti-PD-L1@MMP-gel) demonstrated superior tumor suppression efficacy and prolonged the survival time of the animals with low systemic toxicity. Meanwhile, the OXA&anti-PD-L1@MMP-gel induced an increase in CD8^+^ T cells and M1 macrophages within tumors, and a decrease in Treg cells and M2 macrophages, demonstrating that the drug-loaded system enhanced the antitumor immune response. Moreover, the OXA&anti-PD-L1@MMP-gel effectively inhibited the growth of distal tumors in a bilateral-tumor experiment. **Conclusions:** Consequently, the responsive hydrogel-based chemo-immunotherapy holds potential in tumor treatment.

## 1. Introduction

Cancer continues to pose a significant threat to human health, and formulating effective strategies to inhibit their occurrence and development remains an urgent task [1]. Although traditional treatment methods, such as surgery, chemotherapy, and radiotherapy, have shown some efficacy in clinical oncology, they are plagued by problems such as tumor recurrence and severe toxic side effects [2,3]. Immunotherapy, which leverages the body’s own immune system to enhance the patient’s immunity and eliminate tumor cells, has received significant attention in recent years, owing to its potential to deliver persistent, adaptable, and targeted defense against cancer [4,5]. Immune-checkpoint blockade (ICB) therapy is one of the mainstream tumor immunotherapy methods, which blocks regulatory molecules that tumor cells use to evade immune detection [6,7]. Antibodies targeting cytotoxic T-lymphocyte-associated protein 4 (CTLA-4) and programmed cell death protein 1 (PD-1) and its ligand (PD-L1) have been approved for clinical treatment [8]. However, ICB therapy is hindered by the low objective response rates and treatment-induced immune-related adverse events, prompting further exploration of its improvement and combination with other therapies [9,10].

A promising discovery, though, has emerged: specific chemotherapeutic drugs have the ability to trigger immunogenic cell death (ICD) in cancer cells. During the process, dying tumor cells release damage-associated molecular patterns (DAMPs, such as ATP, CRT and HMGB-1), as well as tumor-associated antigens (TAAs). These substances can activate dendritic cells (DCs) and prime cytotoxic CD8^+^ T cells [11,12]. Chemotherapeutics such as doxorubicin (DOX), oxaliplatin (OXA), bleomycin, bortezomib, and cyclophosphamide fall into this category, and they hold potential for combination with cancer immunotherapy, particularly ICB therapy [13,14,15]. Driven by extensive preclinical studies on ICD-inducing chemotherapeutics, a large number of clinical trials are currently underway, focusing on combinatorial strategies that pair ICD inducers with checkpoint inhibitors. Nevertheless, the safety of such combination therapies remains a key concern in clinical trials, as some regimens may lead to a further escalation of side effects [16,17].

Localized delivery systems offer a promising solution to address these challenges. They are notable for sustained drug release at lesions, reducing systemic doses and adverse reactions. Among them, injectable hydrogels have garnered significant attention due to their ability to provide controlled and prolonged release of therapeutic agents, which is crucial for maintaining the effective concentration of drugs in the tumor microenvironment (TME) [18,19,20,21,22]. In our previous study, we developed an in situ-forming hydrogel via copper-free strain-promoted azide–alkyne cycloaddition (SPAAC) between dibenzocyclooctyne (DBCO)-functionalized poly(L-glutamic acid) (PLG) and azide-modified PLG [23]. This metal-free click reaction crosslinked hydrogel enables rapid gelation under physiological conditions, exhibits high biocompatibility, and facilitates the maintenance of loaded reagent activity, though it still suffers from limited tunability in degradation properties.

In particular, stimulus-responsive hydrogels that degrade in response to tumor-specific cues offer enhanced spatial and temporal control over drug release [24]. Matrix metalloproteinase (MMP)-responsive hydrogels represent a class of such “smart” carriers [25]. MMPs constitute a family of zinc-dependent endopeptidases, which play a crucial role in the remodeling of the extracellular matrix (ECM) and are often overexpressed in the TME, thereby promoting tumor invasion, angiogenesis, and metastasis [26,27]. Among them, MMP-2 and MMP-9 are two key subtypes that target type IV collagen (the major component of the basement membrane) and gelatin (denatured collagen), and are considered as promising triggering factors for targeted drug delivery due to their specific overexpression in various malignant tumors [28,29]. Hydrogels responsive to MMPs typically achieve selective recognition and response to specific MMPs (such as MMP-2 and MMP-9) through a designed rational crosslinking network [30]. Unlike conventional hydrogels that rely on non-degradable or passive degradation crosslinking structures, these hydrogels incorporate MMP-cleavable peptide sequences into their networks. Commonly used sequences include PLGVR, PVGLIG, and PLGLAG, which originate from natural MMP substrates, such as collagen [31,32,33]. These peptides, upon recognition and hydrolysis by MMPs at specific cleavage sites, can disrupt the integrity of the hydrogel, thereby facilitating the on-demand release of encapsulated therapeutic agents.

**Figure 1:**
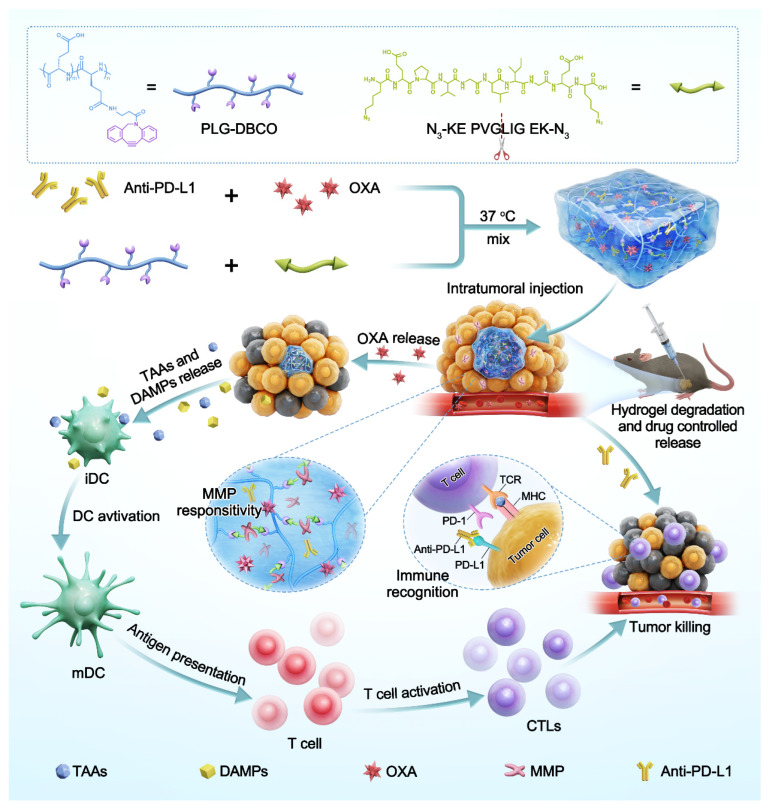
Schematic diagram for the preparation of OXA and anti-PD-L1 co-loaded hydrogel and its application in in situ sustained drug delivery for chemo-immunotherapy.

Accordingly, we engineered an injectable, MMP-responsive hydrogel depot for local chemo-immunotherapy. The hydrogel is formed via copper-free SPAAC between DBCO-functionalized poly(L-glutamic acid) (PLG-DBCO) and an MMP-cleavable peptide crosslinker, N_3_-KEPVGLIGEK-N_3_. This network physically co-encapsulates OXA and the immune-checkpoint inhibitor anti-PD-L1, as illustrated in Figure 1. The MMP-responsive hydrogel was characterized in terms of its mechanical properties, biodegradability, and biocompatibility. The sustained-release profiles of OXA from the hydrogel were examined in PBS, both in the presence and absence of collagenase type IV. The ability of the OXA-laden hydrogel to induce ICD in B16F10 melanoma cells was confirmed. The antitumor effect and systemic toxicity of the OXA/anti-PD-L1 co-loaded hydrogel were evaluated in vivo via intratumoral administration in C57BL/6 mice bearing B16F10 melanoma tumors. The immune microenvironment following treatment was profiled by analyzing infiltrating immune cells and cytokine secretion.

## 2. Materials and Methods

### 2.1. Materials

Azide-modified MMP-cleavable peptide (N_3_-KEPVGLIGEK-N_3_) and γ-Benzyl-L-glutamate were obtained from GL Biochem Ltd. (Shanghai, China). Dibenzocyclooctyne-ethylamine (DBCO-CH_2_CH_2_NH_2_) was purchased from Biomatrik (Jiaxing, China). Azide-modified PEG (N_3_-PEG-N_3_, Mn = 1000 Da) was purchased from Poursure Biotechnology (Shanghai, China). Oxaliplatin (OXA) was obtained from Shandong Boyuan Pharm (Jinan, China). Other chemical reagents were purchased from Sinopharm Chemical Reagent Co., Ltd. (Shanghai, China) and used as obtained.

Cell counting kit-8 (CCK-8) and cell culture media were purchased from Meilunbio (Dalian, China). InVivoMAb anti-mouse PD-L1 (BE0101) was obtained from BioXcell (West Lebanon, IN, USA). Cy3-IgG (A10522), Cy5-IgG (A10525), and ELISA kits for TNF-α (88-7324-88) and IFN-γ (88-7314-88) were purchased from Thermo Fisher (San Jose, CA, USA). Collagenase type IV (A004186) was obtained from Sangon Biotech (Shanghai, China). Anti-Calreticulin antibody (ab196159) and anti-HMGB1 antibody (ab195010) were purchased from Abcam (Waltham, MA, USA). Other antibodies used in flow cytometry were obtained from BioLegend (San Diego, CA, USA).

B16F10 melanoma and NIH 3T3 mouse fibroblast cell lines were obtained from ATCC (CRL-6475 and CRL-1658) and maintained according to ATCC guidelines.

### 2.2. Synthesis and Characterization of Poly(L-Glutamic Acid) Functionalized with Dibenzocyclooctyne (PLG-DBCO)

PLG-DBCO was prepared according to the method previously reported [23]. γ-benzyl-L-glutamate *N*-carboxyanhydride (BLG NCA) (10 g) was dissolved in 200 mL of anhydrous DMF, and n-hexylamine was added as an initiator at specific molar amounts to control the degree of polymerization (DP) (0.38 mmol for DP = 100, 0.19 mmol for DP = 200, 0.127 mmol for DP = 300). The mixture reacted under vacuum for 3 days, then 1 mL acetic anhydride was added for 12 h to terminate. After concentrating to 50% volume at 50 °C under reduced pressure conditions, it was precipitated in ice-cold ether, filtered, and dried to yield poly(γ-benzyl-L-glutamate) (PBLG).

PBLG was dissolved in 69 mL dichloroacetic acid, deprotected with 23 mL 33 wt% HBr/acetic acid at room temperature for 2 h, then precipitated in cold ether. The solid was redissolved in anhydrous 60 mL of DMF, dialyzed (MWCO 3.5 kDa), and lyophilized to afford PLG.

PLG (1 g) was dissolved in 60 mL of anhydrous DMF at 60 °C [23]. After complete dissolution, EDC·HCl (300 mg) and NHS (175 mg) were added, and the mixture was stirred at room temperature for 30 min. Subsequently, DBCO-CH_2_CH_2_NH_2_ (200 mg) was added, and the reaction was allowed to proceed for 3 days under light-protected conditions. The resulting product was dialyzed for 3 days and lyophilized to obtain PLG-DBCO.

The chemical structures of the products were characterized by ^1^H NMR spectroscopy (Bruker AV 500 MHz). The molecular weights and the distributions of these polymers were determined by gel permeation chromatography (GPC).

### 2.3. Preparation and Characterization of the Hydrogel

For the preparation of the MMP-responsive hydrogel (MMP-gel), N_3_-KEPVGLIGEK-N_3_ was dissolved in PBS (pH 7.4) to prepare solutions with concentrations of 3%, 4%, 5%, 6% *w*/*v*, respectively. Correspondingly, PLG-DBCO was dissolved in PBS to obtain solutions of the same concentrations. The two types of solutions (with matching concentrations) were mixed at an N_3_/DBCO molar ratio of 1:1 in a vial and incubated in a water bath at 37 °C. Successful gelation was confirmed by vial inversion, wherein the formation of a stable, non-flowing material indicated the formation of MMP-gel. The PEG-gel was prepared via the same procedure as the MMP-gel by substituting N_3_-KEPVGLIGEK-N_3_ with N_3_-PEG-N_3_.

The morphology of both hydrogels (MMP-gel and PEG-gel) were examined with a ZEISS Sigma 300 cryo-SEM. Their mechanical properties were characterized on an Anton Paar MCR 301 rheometer fitted with a 25 mm parallel plate, testing at 37 °C with a fixed gap of 0.5 mm. The rheological measurement was performed following earlier studies [34].

### 2.4. Degradation Behavior of the Hydrogel

The in vitro degradation of hydrogels was assessed by monitoring mass loss over time. Precursor solutions (5% *w*/*v*) of PLG-DBCO and the corresponding crosslinker (N_3_-KEPVGLIGEK-N_3_ for MMP-gel or N_3_-PEG-N_3_ for PEG-gel) were prepared into pre-weighed vials (100 µL). After gelation at 37 °C, the initial mass was recorded. Samples were incubated at 37 °C in PBS with or without collagenase type IV (1 U/mL) under constant shaking at 60 rpm. At designated time points, the remaining hydrogel mass was measured, and the mass-remaining percentage was calculated. Fresh medium was replenished after each measurement.

The in vivo degradation profile of the hydrogel was evaluated in female C57BL/6 mice (6–8 weeks old). A total of 100 µL of precursor solution (5% *w*/*v*) was subcutaneously injected into the dorsal region of each mouse. At predetermined time points, the mice were euthanized and dissected. The residual hydrogel was photographed, excised, and weighed. Perilesional skin tissues were collected for H&E staining after fixation (4% paraformaldehyde (PFA)) and embedding.

### 2.5. Drug Release

#### 2.5.1. In Vitro Release of OXA

A stock solution of OXA was prepared in sterile water. PLG-DBCO was dissolved in the OXA solution to form a 5% (*w*/*v*) precursor, while N_3_-KEPVGLIGEK-N_3_ and N_3_-PEG-N_3_ were separately dissolved in PBS. The solutions were combined in vials (200 μL per vial) with a final OXA concentration of 1 mg/mL and incubated at 37 °C for 1 h to form MMP-gel and PEG-gel. Subsequently, PBS or PBS containing collagenase type IV (1 U/mL) was added to each vial (1.5 mL). At predetermined intervals, the release medium was harvested and substituted with fresh medium, and the harvested samples were digested with ultrapure HNO_3_ at 130 °C until the solution became clear. The digests were diluted until the Pt content was less than 0.1 μg/mL, and the Pt concentration was analyzed by inductively coupled plasma mass spectrometry (ICP-MS).

#### 2.5.2. In Vitro Release of Cy3-IgG

The release of Cy3-IgG was conducted following a procedure like that of OXA. Each vial received 200 μL of hydrogel precursor solution containing Cy3-IgG at a final concentration of 0.5 mg/mL. After gelation, release medium was added, and samples were periodically taken with medium replacement. Cy3-IgG concentrations were determined by UV spectrophotometry against a standard curve.

#### 2.5.3. In Vivo Release of Cy5-IgG

Female C57BL/6 mice (6–8 weeks old) were randomly divided into three groups and administered a subcutaneous injection (100 µL per mouse) of the following: (A) PBS containing 0.5 mg/mL of Cy5-IgG; (B) MMP-gel precursor solution with 0.5 mg/mL of Cy5-IgG; or (C) PEG-gel precursor solution with 0.5 mg/mL of Cy5-IgG. The release and distribution of Cy5-IgG were tracked in vivo using an IVIS Lumina LT imaging system, with real-time fluorescence imaging to assess the release kinetics from hydrogels.

### 2.6. Material Cytotoxicity

Biocompatibility was evaluated using a cytotoxicity assay with NIH 3T3 mouse embryonic fibroblasts. Cells were seeded at 7000 cells per well in 96-well plates and cultured for 24 h. Each hydrogel component was prepared as a 10 mg/mL stock and serially diluted in complete medium to concentrations ranging from 0.5 to 0.015625 mg/mL. After aspirating the culture medium, 100 µL of each dilution was added and incubated for 24 h. The material-containing medium was then removed, and 100 µL of CCK-8 reagent (10% *v*/*v* in complete medium) was added. After incubation for 2 h, the absorbance was measured at 450 nm using a Tecan Spark microplate reader.

### 2.7. Immunogenic Cell Death (ICD) Induction

#### 2.7.1. Exposure of Calreticulin (CRT)

B16F10 cells (3 × 10^5^/well) were seeded in 6-well plates and cultured for 24 h. Treatments included PBS, blank MMP-gel, free OXA, and OXA@MMP-gel (10 µL each). After 4 h, cells were detached using trypsin-EDTA, collected, and washed. Samples were stained with Alexa Fluor 647-conjugated anti-CRT antibody for 1 h at room temperature in the dark, then washed and analyzed by flow cytometry (BD FACSCelesta, BD Biosciences, San Jose, CA, USA).

#### 2.7.2. Release of HMGB-1

B16F10 cells (1.5 × 10^5^/well) were cultured for 24 h. The same four formulations were applied for 24 h. Cells were then detached, fixed/permeabilized, and stained intracellularly with Alexa Fluor 488-conjugated anti-HMGB-1 antibody for 30 min. After washing, HMGB-1 levels were assessed via flow cytometry.

### 2.8. B16F10 Unilateral Tumor Model

The B16F10 unilateral tumor model was established by subcutaneous injection of 1 × 10^6^ cells into the right flank of female C57BL/6 mice. The tumor volume was calculated using Equation (1).

(1) $$\mathrm{Volume}=\frac{\mathrm{Length}\times {\mathrm{Width}}^{2}}{2}$$

When the tumors reached approximately 50 mm^3^, the mice were divided into six groups randomly and treated via intratumoral injection with the following formulations at 50 μL for each mouse (n = 5): (A): PBS; (B): Free OXA&anti-PD-L1; (C): OXA@MMP-gel; (D): anti-PD-L1@MMP-gel; (E): OXA&anti-PD-L1@PEG-gel; (F): OXA&anti-PD-L1@MMP-gel. The injection dosages for OXA and anti-PD-L1 were 3 mg/kg and 2 mg/kg, respectively. Tumor volume and body weight were recorded every two days. When tumor volumes in the PBS group exceeded 1500 mm^3^, all mice were euthanized. A mouse was randomly selected from each group, and its heart, liver, spleen, lung, and kidney were collected and fixed in 4% PFA for subsequent sectioning and H&E staining. The same experimental procedure was followed to monitor the survival of the mice separately (n = 5). The mice were euthanized once their tumor volumes surpassed 1500 mm^3^.

### 2.9. Immune Cell Analysis of the B16F10 Unilateral Tumor Model

Bilateral lymph nodes were collected in a 24-well plate containing 1 mL cold FACS buffer (PBS with 2% FBS), mechanically dissociated using a syringe plunger, filtered, and centrifuged at 4 °C (1500 rpm, 5 min). The pellets were resuspended in fresh buffer, centrifuged again, and stained with 100 μL antibody cocktail (PE-CD11c, APC-CD80, PE/Cy7-CD86) for 45 min at 4 °C in the dark. After the cells were washed, they were fixed in 4% PFA and analyzed by flow cytometry.

Tumors were weighed, minced, and digested in FACS buffer, filtered, and centrifuged. Cell pellets were resuspended and divided into four groups for subset staining: T cells (FITC-CD3/PE-CD4/APC-CD8a), DCs (PE-CD11c/APC-CD80/APC/Cy7-MHC II), macrophages (FITC-CD11b/PE/Cy7-CD206/APC-CD80), and Tregs (FITC-CD3/PE/Cy7-CD4/PE-CD25/APC-Foxp3). All groups were incubated with antibodies in a dark environment for 45 min at 4 °C, then washed and fixed in 4% PFA for analysis. Treg samples required additional steps: permeabilization with fixative, intracellular staining with Foxp3 antibody, and washing before fixation.

The spleens were dissociated in FACS buffer, filtered, and centrifuged. Cells were stained with surface antibodies (FITC-CD3, PE-CD4, APC-CD8a) for 30 min on ice, washed, fixed, permeabilized, and intracellularly stained with PE-IFN-γ. After the cells were washed, they were resuspended in 4% PFA for flow cytometry. Antibody staining and flow cytometry procedures were performed following the methods described in previous studies [35].

The levels of TNF-α and IFN-γ in tumor tissues were measured using mouse ELISA kits according to manufacturer instructions. The absorbance was measured at 450 nm using a Tecan Spark microplate reader.

### 2.10. Bilateral-Tumor Model

To evaluate systemic immune responses induced by local chemo-immunotherapy, a bilateral B16F10 melanoma model was established in female C57BL/6 mice (6–8 weeks old). First, the tumor cells (1 × 10^6^) in 50 µL PBS were injected subcutaneously into the right flank to form the proximal tumor. After 48 hours, the same number of cells were injected into the left flank to establish the distal tumor. Treatment began when the proximal tumor reached ~50 mm^3^. Mice were randomly divided into six groups and treated via intratumoral injection of proximal tumor with formulations the same as that in the B16F10 tumor model. Tumor volume and body weight were recorded every two days. The mice were euthanized when tumor volumes in the PBS group exceeded 1500 mm^3^. The proximal tumors were fixed for H&E, TUNEL, and immunofluorescence staining. The distal tumors were analyzed by flow cytometry for immune cell infiltration (T cells, DCs, macrophages) and ELISA for cytokine levels (TNF-α, IFN-γ).

### 2.11. Animal Procedure

The female C57BL/6 mice were obtained from Vital River Company (Beijing, China). The animal housing environment was maintained at a constant temperature of 22.0 ± 4.0 °C, and a 12 h light/dark cycle was followed.

### 2.12. Statistical Analysis

All experimental data are presented as mean ± standard deviation (SD). Statistical significance was evaluated by one-way ANOVA followed by LSD post hoc multiple comparisons (n ≥ 3). Mouse survival data were plotted as Kaplan–Meier curves and analyzed using the log-rank test. Statistical significance was defined as * *p* < 0.05, ** *p* < 0.01, *** *p* < 0.001 and ns: no significance.

## 3. Results and Discussion

### 3.1. Preparation and Characterization of Hydrogels

The MMP-responsive hydrogel (MMP-gel) and the control PEG-based hydrogel (PEG-gel) were prepared via the SPAAC reaction between the DBCO of PLG-DBCO and the azide termini of the MMP-cleavable peptide (N_3_-KEPVGLIGEK-N_3_) or the control segment (N_3_-PEG-N_3_), respectively. The synthesis of PLG-DBCO followed a previously established method from our group [23], and the corresponding synthetic route is depicted in Figure S1. The molecular weight distribution of polymers was determined by GPC, and the polydispersity index (PDI) of PLG was found to be 1.57. Successful synthesis of PLG-DBCO was confirmed by ^1^H NMR spectroscopy, with a calculated DBCO grafting ratio of 8% (Figures S2 and S3).

**Figure 2:**
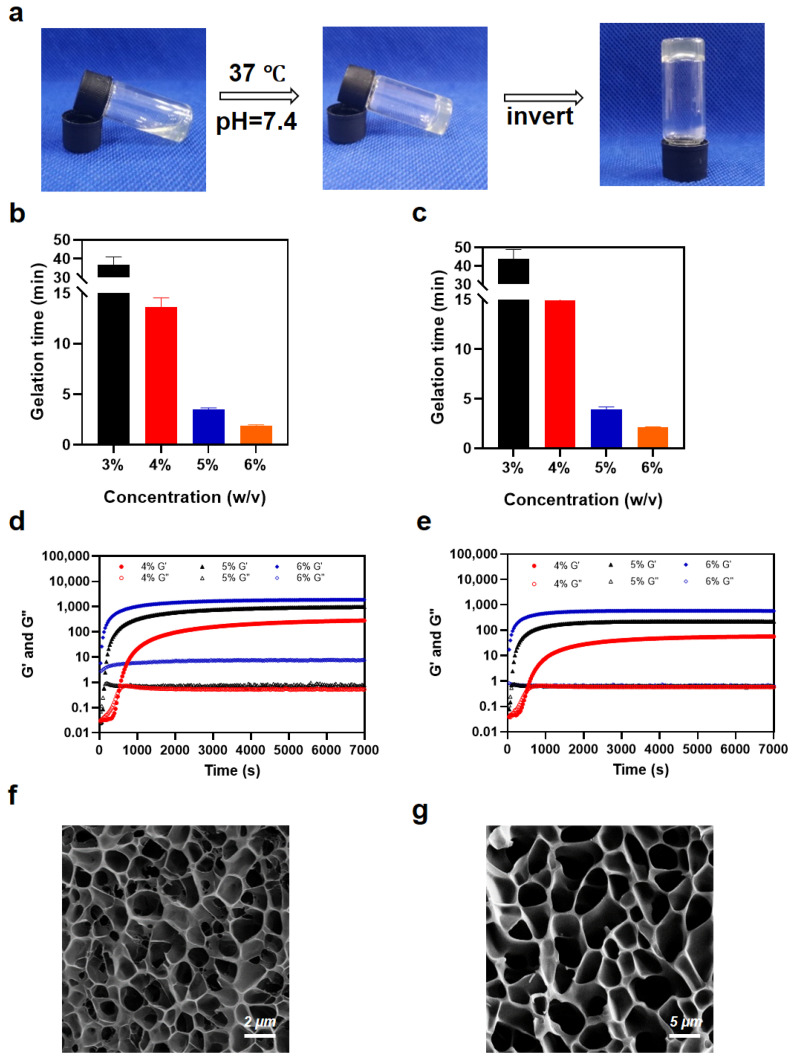
Preparation and characterization of hydrogels. (**a**) Photographs of formation of 5% (*w*/*v*) MMP-gel. Gelation time of (**b**) MMP-gel and (**c**) PEG-gel at different polymer concentrations (n = 3). Rheological results of MMP-gel (**d**) and PEG-gel (**e**) at different polymer concentrations. Cryo-SEM images of 5% (*w*/*v*) MMP-gel (**f**) and PEG-gel (**g**). Data are presented as means ± SD.

The MMP-Gel and PEG-gel were prepared under identical conditions by mixing PLG-DBCO with their respective azide crosslinkers (N_3_/DBCO molar ratio = 1:1) in a vial and incubated at 37 °C (Figure 2a). The measurement results of gelation time revealed that increasing the polymer concentration markedly accelerated the sol–gel transition process (Figure 2b,c). Specifically, raising the polymer concentration from 3% to 6% *w*/*v* shortened the gelation time from 40 min to 2 min.

Rheological analyses corroborated the inverted-vial observations, revealing a concentration-dependent acceleration of gelation. When the polymer concentration increased from 4% to 6% *w*/*v*, the plateau storage modulus (G′) increased from 290 Pa to 1870 Pa, which indicated a significant enhancement in mechanical strength (Figure 2d,e). The MMP-gel exhibited a slightly higher modulus than that of the PEG-gel at the same concentration. Furthermore, the cryo-SEM imaging was performed on 5% *w*/*v* MMP-gel and 5% *w*/*v* PEG-gel. Both hydrogels displayed uniform, highly interconnected three-dimensional networks—architectures favorable for sustained drug delivery. The MMP-gel possessed a markedly denser mesh with pore diameters of ~1–2 µm, whereas PEG-gel exhibited larger pores of ~5–8 µm (Figure 2f,g). This difference in pore size and density accounts for the better mechanical rigidity of MMP-gel and aligns with the rheological data [36]. Considering the gelation time and mechanical properties, the 5% *w*/*v* hydrogel is more suitable for injectable topical drug sustained-release carriers, so it was employed in subsequent experiments.

### 3.2. The Responsive Degradation of Hydrogels

**Figure 3:**
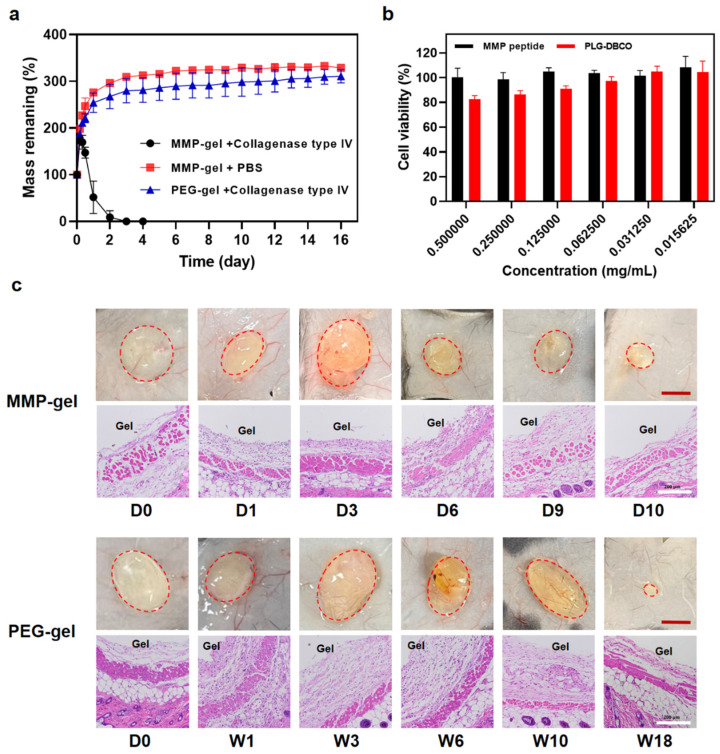
Degradation behaviors and biocompatibility of hydrogels. (**a**) In vitro degradation curves of MMP-gel and PEG-gel (n = 4). (**b**) Viability of NIH 3T3 cells incubated with MMP-cleavable peptide and PLG-DBCO at various concentrations (n = 5) (**c**) In vivo degradation (scale bar = 0.5 cm) and H&E staining (scale bar = 200 μm) of MMP-gel and PEG-gel (n = 4). Data are presented as means ± SD.

The degradation rate is a crucial parameter for evaluating the performance of drug delivery carriers, as it directly affects the efficacy of drug release [20]. In order to investigate the MMP-responsive degradation of MMP-gel, the degradation behavior of both hydrogels in two different degradation media (PBS and PBS containing collagenase type IV (1 U/mL)) was analyzed. The collagenase type IV, also known as gelatinase, includes MMP-2 and MMP-9 [37]. The results showed that the MMP-gel remained stable in PBS for up to 16 days, but in the presence of collagenase type IV, it completely degraded within 3 days (Figure 3a). In contrast, the PEG-gel did not show any significant degradation even after 16 days in the collagenase type IV solution. These findings demonstrate the hydrogel’s MMP-responsive nature, which confers its capacity for degradation within the tumor microenvironment (TME). Meanwhile, the cytotoxicity experiments showed that the viability of NIH 3T3 cells treated with different concentrations of PLG-DBCO and N_3_-KEPVGLIGEK-N_3_ were both higher than 80% compared with the control group (Figure 3b). These results indicate that neither of these two materials has significant cytotoxicity to NIH 3T3 cells, proving that the obtained MMP-gel has good biocompatibility.

To evaluate the in vivo degradation behavior and biocompatibility of the hydrogels, we conducted subcutaneous implantation studies in healthy mice. The results showed that the PEG-gel remained intact within 10 weeks, and there were residual hydrogels that were not completely degraded within 18 weeks. The MMP-gel underwent rapid degradation within 10 days, with a significantly faster degradation rate compared to that of the PEG-gel (Figure 3c). The histological assessment via H&E staining was employed to characterize the inflammatory response in the surrounding tissues of hydrogels. During the first week of MMP-gel injection, a slight elevation in inflammatory cell infiltration was observed, indicative of a mild foreign body reaction. Notably, the number of nearby inflammatory cells decreased, and the inflammatory response gradually eased with the degradation of MMP-gel, and it resolved completely following the full degradation of hydrogels. In addition, no notable signs of necrosis, edema, congestion, or hemorrhage were observed at the injection site throughout the study period. In comparison, the PEG-gel elicited a more pronounced and sustained inflammatory response, correlating with its prolonged degradation. These results indicate that the MMP-gel exhibits favorable biocompatibility and biodegradability under in vivo conditions.

### 3.3. The Responsive Drug Release of Hydrogels

**Figure 4:**
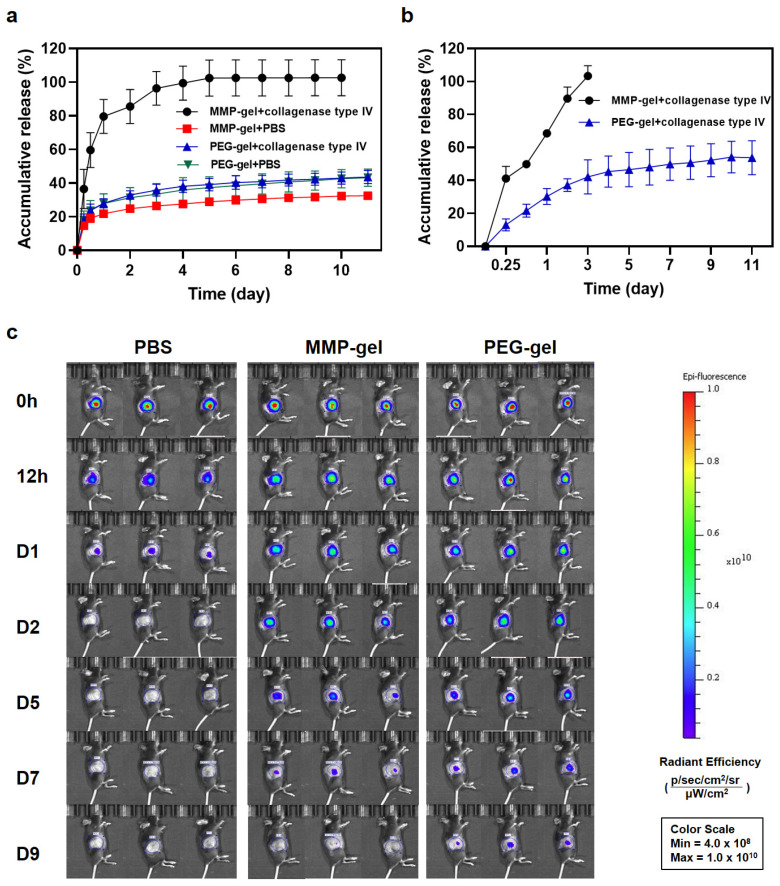
MMP-responsive drug release of hydrogels. (**a**) Release profiles of OXA from OXA@MMP-gel and OXA@PEG-gel (n = 3). (**b**) Release profiles of IgG from IgG@MMP-gel and IgG@PEG-gel (n = 3). (**c**) In vivo real-time NIR fluorescence imaging for release kinetics of Cy5-labeled IgG. Data are presented as means ± SD.

The in vivo and in vitro degradation experiments have shown that MMP-gel has the characteristics of promoting degradation by MMP, indicating its potential as a sustained-release drug carrier. Subsequent experiments were conducted to evaluate the collagenase type IV-induced release behavior of agents in hydrogels. The in vitro release experiment of OXA showed that under the action of collagenase type IV, the OXA in the MMP-gel completely released within 3 days. However, the release amount of the MMP-gel in PBS was only 30%. In contrast, the release of OXA from the PEG-gel did not show significant differences in the two media. Following 11 days of incubation, the cumulative release was measured at 42% in the collagenase solution and 43% in PBS, respectively, as shown in Figure 4a. These results demonstrate that the MMP-gel possesses efficient responsive OXA release capability, supporting its application as an OXA delivery carrier.

Subsequently, the release of anti-PD-L1 in the hydrogel was simulated using the Cy3-labeled IgG (Cy3-IgG) as a model antibody. As illustrated in Figure 4b, the MMP-gel enabled sustained release of IgG under the action of collagenase type IV, achieving complete release within 3 days. In contrast, the PEG-gel released only 50% of the payload after 11 days under the same enzymatic condition. The drug release kinetics of the MMP-gel in collagenase type IV was correlated closely with its in vitro degradation, further supporting its suitability as a sustained-release drug carrier for both OXA and anti-PD-L1.

In order to investigate the release profile of anti-PD-L1 in vivo, Cy5-labeled IgG (Cy5-IgG) was used as a model to simulate its release behavior. The in vivo imaging results showed that the IgG in the PBS group was completely released within 2 days, while the MMP-gel group could achieve a long-lasting sustained release of 7 days, which corresponds to the in vivo degradation results of the hydrogel. The PEG-gel had a slower drug release rate and a longer release duration as shown in Figure 4c, which may be attributed to its lack of MMP responsiveness. These in vivo release results of Cy5-IgG demonstrated that the MMP-gel has a sustained-release effect on IgG, and thus can serve as a sustained-release carrier for anti-PD-L1.

### 3.4. Induction of Immunogenic Cell Death

**Figure 5:**
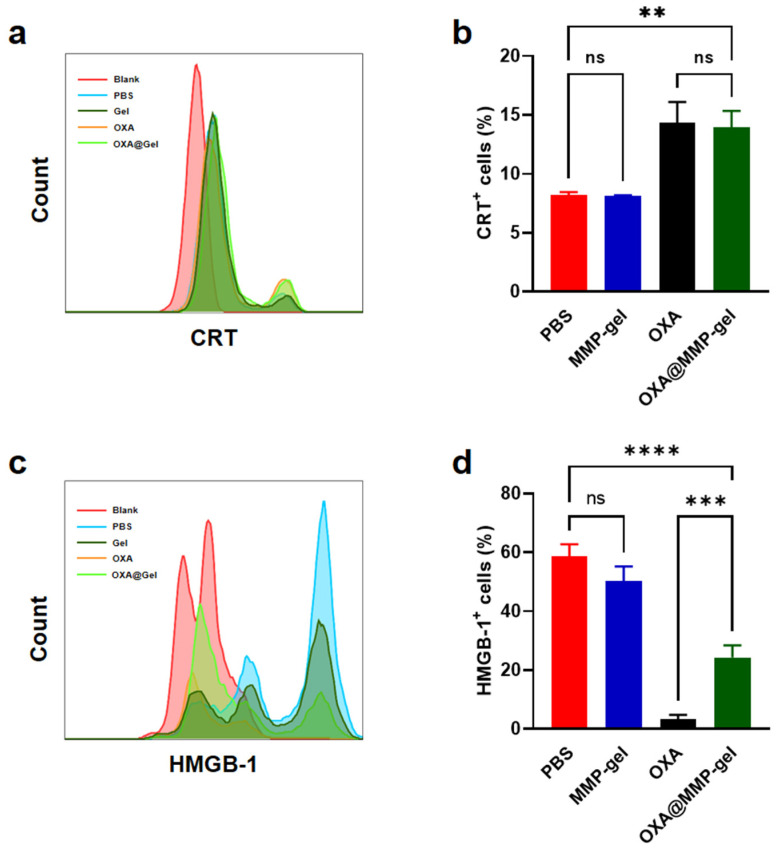
Immunogenic cell death induced by OXA@MMP-gel. (**a**) Representative flow-cytometric analysis of CRT exposure. (**b**) Quantitative analysis of CRT surface expression (n = 3). (**c**) Representative flow-cytometric assessment of HMGB-1 release. (**d**) Quantitative analysis of intracellular HMGB-1 levels (n = 3). Data are presented as means ± SD. Statistical significance was determined by one-way ANOVA followed by LSD post hoc multiple comparisons (** *p* < 0.01, *** *p* < 0.001, **** *p* < 0.0001, ns represents no significance).

Based on previous studies demonstrating that the OXA can induce ICD accompanied by the release of DAMPs, this study evaluated ICD effect mediated by free OXA and OXA-loaded hydrogel (OXA@MMP-Gel) by detecting two central ICD biomarkers: the exposure of calreticulin (CRT) and the translocation of intracellular high-mobility group box 1 (HMGB-1) [14,15]. The CRT detection results showed that the CRT expression levels in the blank hydrogel group and the PBS group were relatively low, with no significant difference. However, the CRT expression levels in the free OXA group and the OXA@MMP-Gel group were higher and at the same level (Figure 5a,b). This phenomenon indicates that both of free OXA and OXA@MMP-Gel can induce ICD in B16F10 cells. It was found that there was no significant difference in the intracellular HMGB-1 content between the blank hydrogel group and the PBS group, while the content in the free OXA group and the OXA@MMP-Gel group was significantly reduced (Figure 5c,d). This indicates that both can induce ICD in B16F10 cells by promoting the massive release of HMGB-1. It is noteworthy that compared with the free OXA group, the intracellular HMGB-1 content in the OXA@MMP-Gel group was relatively higher, and this difference may be attributed to the sustained-drug-release effect of the hydrogel on OXA. In conclusion, these results indicate that by encapsulating the chemotherapy drug OXA in the hydrogel to prepare OXA@MMP-Gel, it can still effectively promote ICD in B16F10 tumor cells, while having the safety advantage of sustained drug release.

### 3.5. Tumor Inhibition Efficacy of OXA and Anti-PD-L1 Co-Loaded Hydrogels in the Unilateral B16F10 Melanoma Model

**Figure 6:**
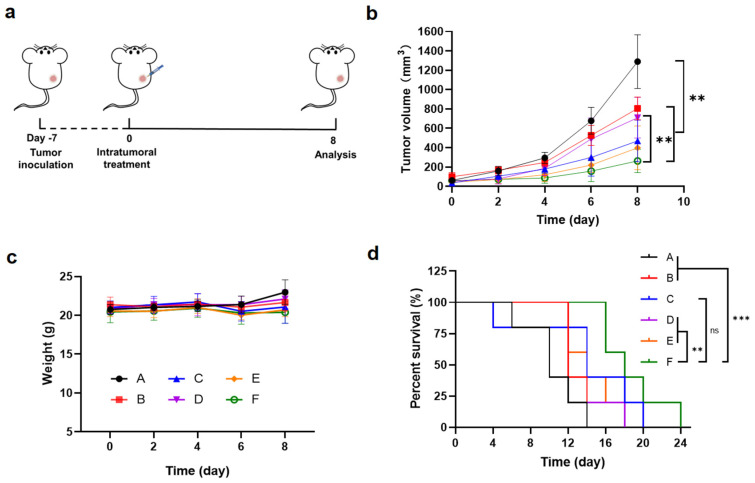
Therapeutic efficacy in the unilateral B16F10 melanoma model. (**a**) Schematic representation of the tumor treatment using chemo-immunotherapy hydrogel. (**b**) Tumor volume progression under indicated treatment regimens (n = 5). (**c**) Body-weight monitoring curves (n = 5). (**d**) Survival curves of mice in different treatment groups (n = 5). (A): PBS; (B): Free OXA&anti-PD-L1; (C): OXA@MMP-gel; (D): anti-PD-L1@MMP-gel; (E): OXA&anti-PD-L1@PEG-gel; (F): OXA&anti-PD-L1@MMP-gel. Data are presented as means ± SD. Statistical analysis was determined by one-way ANOVA followed by LSD post hoc multiple comparisons for (**b**,**c**) and log-rank test for (**d**) (** *p* < 0.01, *** *p* < 0.001, ns represents no significance).

To evaluate the antitumor efficacy of the drug-loaded hydrogel in combination with immunotherapy in vivo, a subcutaneous B16F10 melanoma model was established in female C57BL/6 mice (Figure 6a). When tumor volumes reached approximately 50 mm^3^, the mice were randomly divided into six groups (n = 5). The tumor volume was calculated using Equation (1). The groups were designated as follows: (A): PBS group; (B): Free OXA&anti-PD-L1 group; (C): OXA@MMP-gel group; (D): anti-PD-L1@MMP-gel group; (E): OXA&anti-PD-L1@PEG-gel group; (F): OXA&anti-PD-L1@MMP-gel group. The tumor volume and body weight were monitored to evaluate the therapeutic efficacy and safety profile of the treatments. All the treatment groups significantly inhibited the growth of tumors compared to the PBS control group (Figure 6b and Figure S4). Among them, the hydrogel therapy containing OXA demonstrated a more remarkable antitumor effect compared to free OXA. This is probably attributed to the sustained-release property of hydrogel, which can prevent the rapid diffusion and clearance of the drug, and improve drug stability and local concentration in tumors. Meanwhile, the groups treated with hydrogel co-loaded with both OXA and anti-PD-L1 showed better efficacy than those receiving single-agent formulations, highlighting the advantage of combination therapy in antitumor treatment. Furthermore, the OXA&anti-PD-L1@MMP-gel group exhibited the strongest tumor suppression and extended the survival time that was significantly longer than all other groups (Figure 6d). These results demonstrate that the OXA&anti-PD-L1@MMP-gel treatment leverages its responsive release capability to enhance its therapeutic effect while minimizing systemic toxicity. These results are consistent with the core advantages of the reported local drug delivery system based on hydrogels: The injectable hydrogel prolongs the retention time of the drug in the tumor site through continuous and controlled drug release, thereby enhancing the efficacy of chemo-immunotherapy. At the same time, its low systemic toxicity significantly extends the survival time of experimental animals and improves their quality of life [38].

In terms of the safety evaluation of the treatment, the results showed that the weight loss of the mice during the experiment did not exceed 20% of their initial weight, and the short-term weight loss did not exceed 15% (Figure 6c). This meets the basic requirements for animal weight changes in tumor treatment safety assessment, indicating that the treatments have low toxicity or no obvious toxicity. Further histological analysis of the main organs of the mice using H&E staining did not reveal pathological alterations or toxic damage in any treatment group (Figure S5).

### 3.6. Antitumor Immune Response in the Unilateral B16F10 Melanoma Model

**Figure 7:**
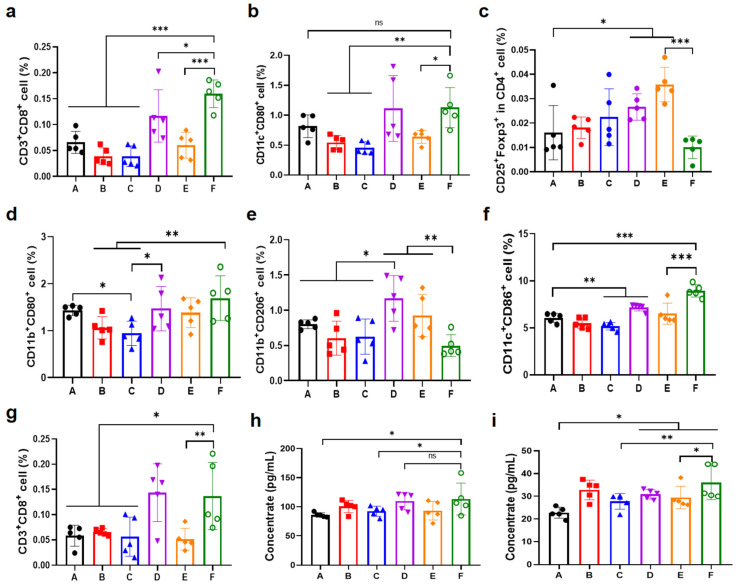
Analysis of immune response at day 8 post-treatment. (**a**) Tumor-infiltrating CD8^+^ T cell populations (n = 5). (**b**) Mature DC populations in tumors (n = 5). (**c**) Treg populations infiltration in tumors (n = 5). (**d**) M1 and (**e**) M2 macrophages in tumors (n = 5). (**f**) Mature DC populations in draining lymph nodes (n = 5). (**g**) CD8^+^ T cell populations in spleen (n = 5). (**h**) IFN-γ and (**i**) TNF-α expression levels in tumors (n = 5). (A): PBS; (B): Free OXA&anti-PD-L1; (C): OXA@MMP-gel; (D): anti-PD-L1@MMP-gel; (E): OXA&anti-PD-L1@PEG-gel; (F): OXA&anti-PD-L1@MMP-gel. Data are presented as means ± SD. Statistical significance was determined by one-way ANOVA followed by LSD post hoc multiple comparisons (* *p* < 0.05, ** *p* < 0.01, *** *p* < 0.001, ns represents no significance).

In order to clarify the regulatory effects of the OXA&anti-PD-L1@MMP-gel system on the tumor immune microenvironment, reveal its mechanism for enhancing tumor immunotherapy efficacy, and verify the advantages of MMP-responsive drug sustained-release vectors, this study detected the proportions of key immune cells in tumor sites, draining lymph nodes, and spleens, and also measured the expression levels of core cytokines in the tumor tissue. The results showed that the OXA&anti-PD-L1@MMP-gel group had significantly increased the infiltration of CD8^+^ T cells and mature DC in tumors compared with other groups (Figure 7a,b), indicating enhanced recruitment of core effector cells mediating tumor-specific immune killing and improved antigen presentation capacity. The increase in CD8^+^ T cell infiltration and the maturation of DCs are key features of effective antitumor immunity. Mature DCs can effectively present tumor antigens to CD8^+^ T cells, thereby activating these cells; and the increase in CD8^+^ T cell infiltration within the tumor is directly related to the improvement of therapeutic effects in tumor models [39]. It was also observed that the polarization of macrophages shifted from the pro-tumor M2 type to the antitumor M1 type, and the number of regulatory T cells (Tregs) decreased of the OXA&anti-PD-L1@MMP-gel group (Figure 7c–e). The reversal of this immunosuppressive microenvironment is consistent with the research results of others, such as those of Wei et al. Their study indicates that the transformation of M2 type macrophages to M1 type and the reduction of Tregs, acting together, can alleviate immunosuppression, thereby enhancing the cytotoxicity mediated by CD8^+^ T cells and improving the efficacy of PD-L1 blocking therapy in “cold tumors” [40]. This indicates that the immunosuppressive state in the tumor microenvironment has been reversed. In addition, the proportion of mature DC in draining lymph nodes and the number of CD8^+^ T cells in the spleen of this group were increased to varying degrees (Figure 7f,g), supporting the initiation of a systemic immune response. Consistent with this, the significant upregulation of two cytokines, IFN-γ and TNF-α (Figure 7h,i), in tumor tissues confirms the activation of the pro-inflammatory pathway. Importantly, there were significant differences in immune cell and cytokine detection indicators between the OXA&anti-PD-L1@MMP-gel group and the OXA&anti-PD-L1@PEG-gel group, which confirmed the critical role of MMP-responsive drug release in enhancing immune activation and therapeutic efficacy.

### 3.7. Chemo-Immunotherapy in a Bilateral-Tumor Model

**Figure 8:**
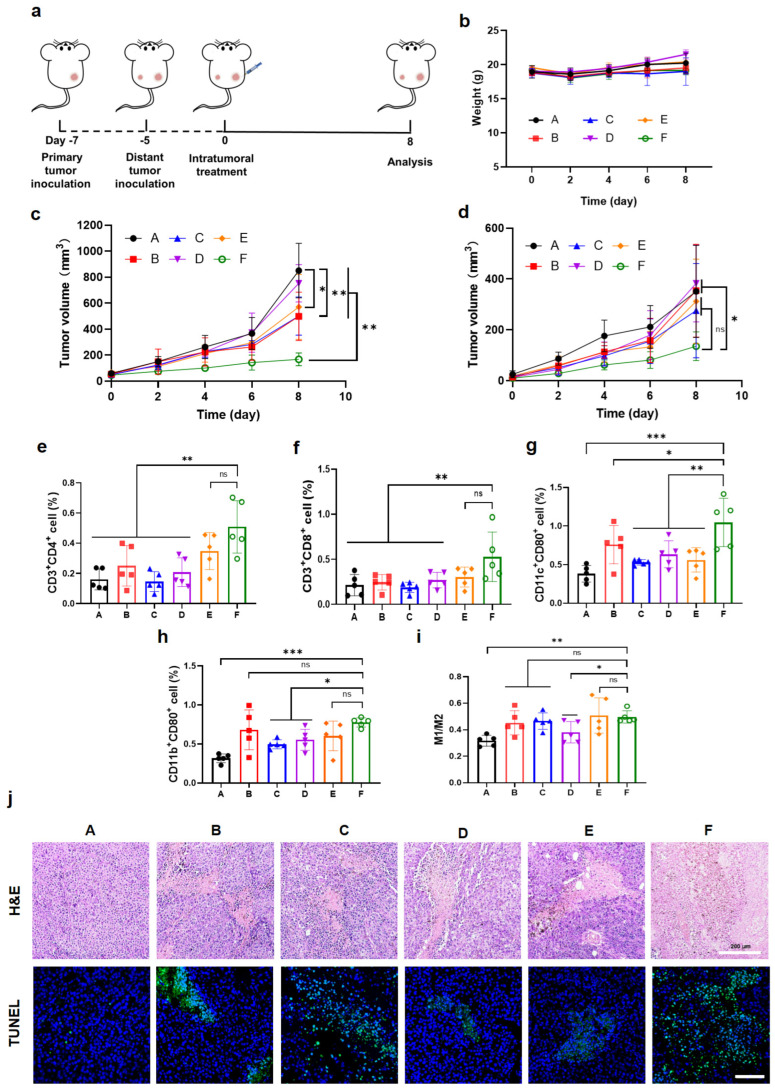
Therapeutic evaluation in the bilateral B16F10 melanoma model. (**a**) Schematic of the bilateral-tumor model. (**b**) Body-weight monitoring curves (n = 5). (**c**) Proximal tumor volume curves for all treatment groups (n = 5). (**d**) Distal tumor volume curves (n = 5). Quantitative analysis of immune cells in the distal tumors: (**e**) CD4^+^ T cell, (**f**) CD8^+^ T cell, (**g**) Mature DCs, (**h**) M1 macrophage, and (**i**) Ratio of M1 to M2 macrophage (n = 5). Histopathology of proximal tumors: (**j**) Representative H&E-stained sections and corresponding TUNEL apoptosis-staining sections for treatment groups A–F, respectively. (A): PBS; (B): Free OXA&anti-PD-L1; (C): OXA@MMP-gel; (D): anti-PD-L1@MMP-gel; (E): OXA&anti-PD-L1@PEG-gel; (F): OXA&anti-PD-L1@MMP-gel. Data are presented as means ± SD. Statistical analysis was determined by one-way ANOVA followed by LSD post hoc multiple comparisons (* *p* < 0.05, ** *p* < 0.01, *** *p* < 0.001, ns represents no significance).

To further validate the systemic antitumor potential and immunomodulatory activity of the combination therapy, the subcutaneous bilateral B16F10 melanoma model was established in female C57BL/6 mice (Figure 8a). The tumor volumes and body weights were dynamically monitored, and histological as well as immunological analyses were performed to elucidate the therapeutic mechanism. It was found that the OXA&anti-PD-L1@MMP-gel group was able to significantly inhibit the growth of both the proximal and distal tumors (Figure 8c,d), and there was no significant decrease in body weight of the mice in each group (Figure 8b). The results of H&E staining and TUNEL assays indicated that the OXA&anti-PD-L1@MMP-gel group had the highest proportion of apoptotic cells compared to other groups (Figure 8j), further demonstrating the superior antitumor efficacy of this treatment.

The immunological analysis of distant tumors indicated that the OXA&anti-PD-L1@MMP-gel group exhibited a significant increase in the infiltration of CD4^+^ T cells and CD8^+^ T cells (Figure 8e,f), along with the increase in the proportion of mature DCs in the tumors (Figure 8g), suggesting the activation of T cells and the presentation of antigens. Furthermore, it was observed that there was a more significant polarization transformation of macrophages in the OXA&anti-PD-L1@MMP-gel group, through the increase in M1 macrophages and the ratio of M1 to M2 macrophages (M1/M2) (Figure 8h,i). These results confirm that this chemo-immunotherapy hydrogel system, which responds to the MMP, can inhibit the growth of local tumors and trigger the systemic immune response to inhibit the spread of distant tumors.

## 4. Conclusions

In response to the high expression of MMPs in tumor cells, this study designed an MMP-responsive injectable hydrogel (MMP-gel) loaded with OXA and anti-PD-L1. This hydrogel was successfully prepared through a copper-free click reaction (SPAAC), using azide-modified MMP-responsive peptide (N_3_-KEPVGLIGEK-N_3_) and DBCO-grafted poly(L-glutamic acid) (PLG-DBCO). The characterization results showed that the MMP-gel had uniform pores, good mechanical properties, and no cytotoxicity to cells. It exhibited MMP-responsive degradation and sustained drug release, achieving complete release of OXA or model IgG within 3 days upon collagenase type IV stimulation, and inducing immunogenic cell death of tumor cells. In vivo studies using unilateral and bilateral melanoma models demonstrated that OXA&anti-PD-L1@MMP-gel could effectively inhibit local and distant tumor growth, trigger systemic antitumor immune responses, alleviate toxic and side effects, and prolong survival. Overall, this MMP-responsive hydrogel can achieve local co-delivery of OXA and anti-PD-L1, providing a promising platform for intelligent chemotherapy–immunotherapy combinations.

## Data Availability

The original contributions presented in this study are included in the article/Supplementary Material. Further inquiries can be directed to the corresponding author.

## Supplementary Materials

The following supporting information can be downloaded at: https://www.mdpi.com/article/10.3390/pharmaceutics17111453/s1, Figure S1: Synthesis route of PLG-DBCO; Figure S2: GPC spectrum for PLG; Figure S3: 1H NMR spectrum of BLG NCA (CDCl3), PBLG (CF3COOD), PLG (D2O) and PLG-DBCO (D2O); Figure S4: (a) Tumor photographs of mice in different groups on day 8. (b) The weights of tumors in different groups on day 8 (n = 5); Figure S5: H&E staining images of heart, liver, spleen, lung, and kidney sections from mice with different treatments.
