# Supplementary material for: Injectable Matrix Metalloproteinase-Responsive Polypeptide Hydrogels as Drug Depots for Antitumor Chemo-Immunotherapy
Source: Pharmaceutics. 2025 Nov 11;17(11):1453. doi: 10.3390/pharmaceutics17111453 (PMC12655423; doi:10.3390/pharmaceutics17111453)
Supplement: Supplementary file 1 [file pharmaceutics-17-01453-s001.zip › pharmaceutics-3944118-supplementary.pdf]

Supplementary Materials：Injectable matrix metalloproteinase-responsive polypeptide hydrogels as drug depots for antitumor chemo-immunotherapy

Shuang Liang , Tianran Wang, Junfeng Ding , Jiaxuan Yang, Chaoliang He, Yan Rong

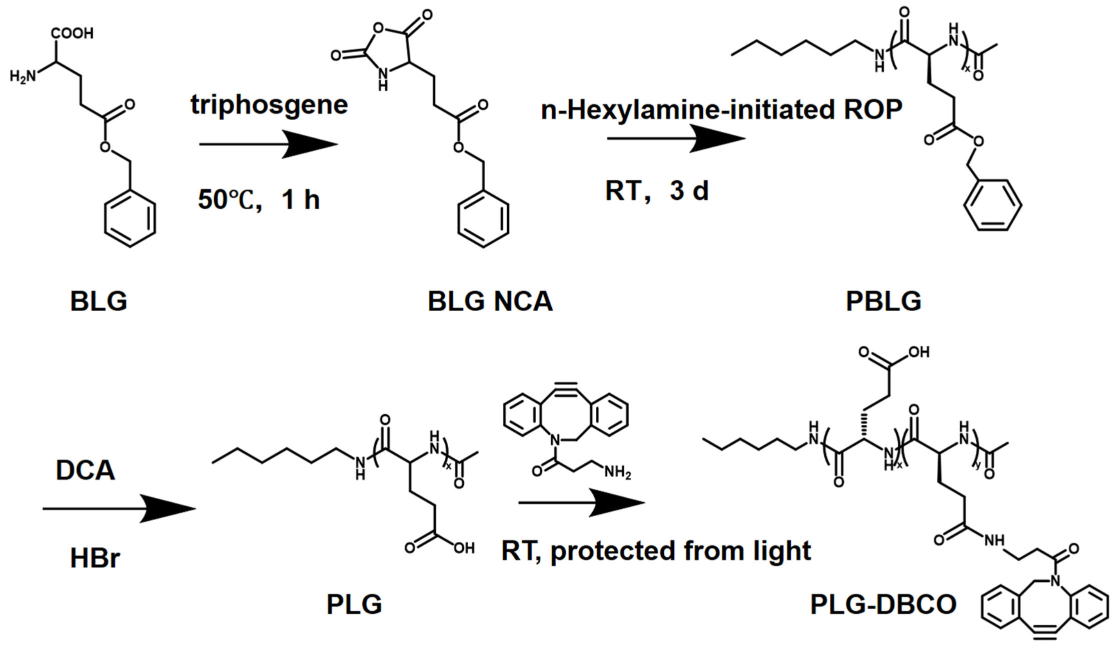

Figure S1. Synthesis route of PLG-DBCO.

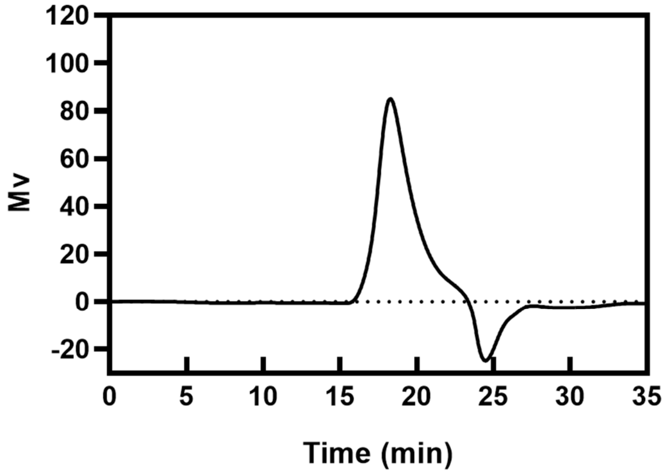

Name	Mw	Mn	PDI
PLG	64900	41100	1.57

Figure S2. GPC spectrum for PLG

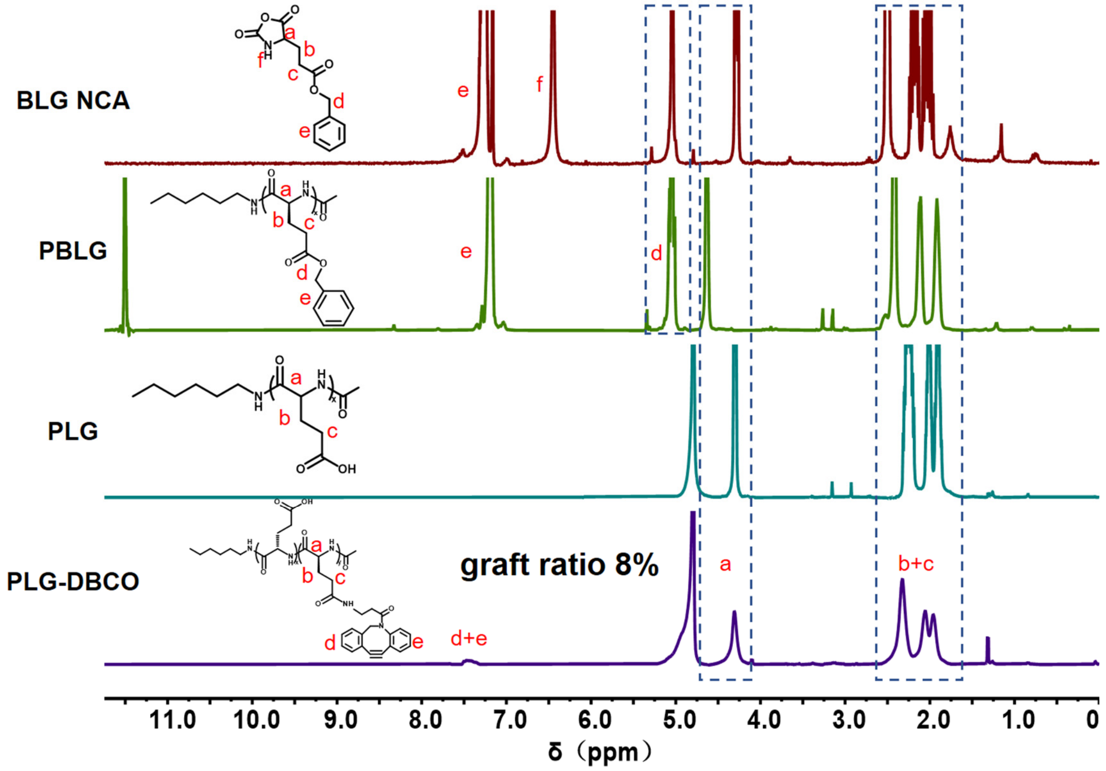

Figure S3. ^{1}H NMR spectrum of BLG NCA ($CDCl_3$), PBLG (CF3COOD), PLG (D_2O) and PLG-DBCO (D_2O).

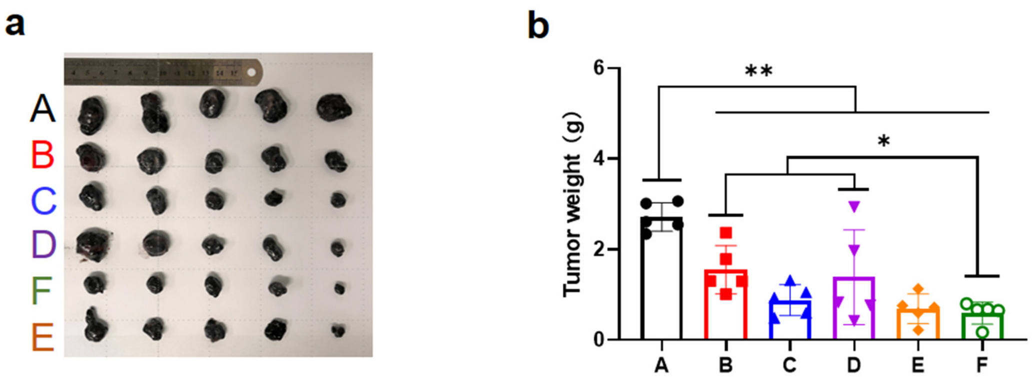

Figure S4. (**a**) Tumor photographs of mice in different groups on day 8. (**b**) The weights of tumors in different groups on day 8 (n = 5). A: PBS; B: Free OXA&anti-PD-L1; C: OXA@MMP-gel; D: anti-PD-L1@MMP-gel; E: OXA&anti-PD-L1@PEG-gel; F: OXA&anti-PD-L1@MMP-gel. Data are presented as the mean ± SD. Statistical significance was analyzed by one-way ANOVA using an LSD posttest.

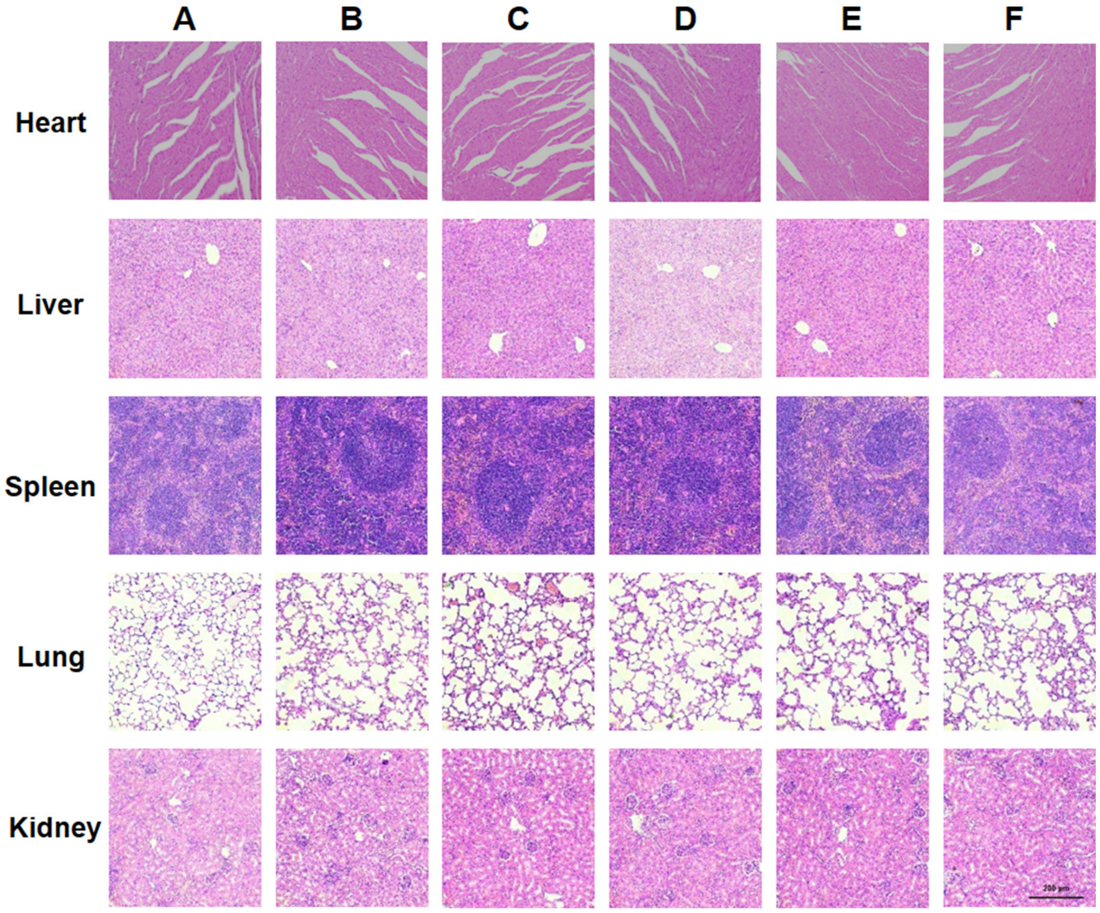

Figure S5. H&E staining images of heart, liver, spleen, lung, and kidney sections from mice with different treatments. Scale bar = 200 μm. A: PBS; B: Free OXA&anti-PD-L1; C: OXA@MMP-gel; D: anti-PD-L1@MMP-gel; E: OXA&anti-PD-L1@PEG-gel; F: OXA&anti-PD-L1@MMP-gel.
